# Clinical Outcomes of a Minimally Invasive Percutaneous Brostrom Technique without Arthroscopic Assistance

**DOI:** 10.3390/diagnostics14192252

**Published:** 2024-10-09

**Authors:** Ettore Vulcano, Gerard F. Marciano, Enrico Pozzessere

**Affiliations:** 1 Mount Sinai Medical Center, Columbia University, Miami, FL 33140, USA; 2 Department of Orthopedics, Columbia University Medical Center, New York-Presbyterian Hospital, New York, NY 10032, USA; 3 Department of Orthopaedics and Traumatology, University of Insubria, Ospedale di Circolo—Fondazione Macchi, 21100 Varese, Italy

**Keywords:** ankle instability, ankle sprain, lateral ligament repair, minimally invasive Brostrom

## Abstract

Background/Objectives: Surgical management of chronic lateral ankle instability has traditionally been performed using an open technique. Arthroscopic-assisted and all-arthroscopic techniques have gained popularity as they have achieved strong clinical outcomes. However, they rely on the surgeon’s arthroscopic skills and familiarity with arthroscopic anatomy. Recently, a minimally invasive percutaneous technique without arthroscopic assistance has been developed that incorporates the benefits of arthroscopy, such as minimal soft tissue disruption, without the additional requirements of performing an arthroscopic technique. The aim of the current study is to describe the minimally invasive percutaneous technique for chronic lateral ankle instability and report on its clinical outcomes. Methods: Fifty-four consecutive patients without intra-articular ankle pathology underwent lateral ligament repair for chronic ankle instability with a percutaneous technique at a single institution by a fellowship-trained foot and ankle surgeon. Foot Function Index (FFI) score was recorded pre-operatively and post-operatively at final follow-up. All patients had a minimum follow-up of 12 months. Post-operative complications and patient satisfaction were also recorded. Results: A significant improvement (*p* < 0.001) in FFI compared to pre-operative values (from 55, SD 4.1, to 10, SD 1.9) was observed. A single patient required a return to the operating room for open revision with allograft reconstruction following a fall 2.5 months post-operatively. There were no other complications including infection or nerve injury. The overall rate of satisfaction after surgery was 98.1%, with one patient dissatisfied due to excessive ankle stiffness. Conclusions: The described minimally invasive percutaneous Brostrom procedure is safe and effective for the treatment of chronic lateral ankle instability without intra-articular ankle pathology.

## 1. Introduction

Ankle sprains constitute 7–10% of all admissions to hospital emergency departments and account for approximately 70% of all ankle injuries [1,2]. The anterior talofibular ligament (ATFL) is the most commonly injured ligament, occurring in 85% of cases, followed by the calcaneofibular ligament (CFL), which is involved 50–75% of the time. Rarely, the posterior talofibular ligament is injured (fewer than 10% of ankle sprains) [3]. Acute ankle sprains can often be managed conservatively; however, in 10–40% of individuals, chronic ankle instability can develop, requiring surgical stabilization [4]. Surgical management can be broadly categorized as anatomic or non-anatomic with repair or reconstruction of the ATFL and/or CFL. Anatomic repair, such as the open Brostrom–Gould procedure, is considered the gold standard and is associated with better functional outcomes and less secondary osteoarthritis when compared to non-anatomic repair [5,6].

Clinical results for arthroscopic techniques have been excellent [7], and similar results have been achieved arthroscopically compared to those achieved with the open Brostrom procedure [8,9]. Arthroscopic techniques have been described by multiple authors, with many recommending arthroscopy prior to any lateral ligament procedure, regardless of technique, to investigate intra-articular pathology [10,11,12,13,14]. Arthroscopic techniques can be completed with only arthroscopy (all-inside) or arthroscopic assisted techniques (percutaneous) [15,16,17]. Notably, arthroscopic repair has demonstrated faster return to sport and improved early functional outcomes compared to open techniques [18]. The theoretical benefits of arthroscopic techniques are less soft tissue disruption in an area with minimal subcutaneous tissue and the ability to simultaneously identify and address intra-articular pathologies.

Despite increasing arthroscopic ankle procedures, exposure of orthopedic residents to arthroscopic ankle procedures remains relatively low and highly variable [19]. Thus, an all percutaneous technique without any arthroscopic assistance was devised to reap the theoretical benefits of arthroscopic techniques without necessitating advanced arthroscopic skills. The aim of this study is to describe this minimally invasive technique for lateral ankle instability and report the clinical outcomes of patients undergoing the procedure.

## 2. Materials and Methods

A retrospective medical record review was conducted on 54 consecutive patients who underwent a minimally invasive percutaneous Brostrom procedure from September 2016 to August 2022. Patients were included if they had symptomatic chronic ankle instability for greater than 6 months with at least one-year follow-up from their procedure. Chronic ankle instability included both mechanical and functional instability. Mechanical instability was defined by a positive anterior drawer sign and a positive talar tilt test. Functional instability was defined by patient-reported symptoms, such as a sense of the ankle giving way (without significant clinic ligamentous laxity), proprioceptive deficits, or a feeling of muscle weakness. Conservative treatment was considered unsuccessful after a minimum of 3 months of targeted physical therapy focusing on joint mobility exercises, Achilles tendon stretching, and strengthening of the dorsiflexors and peroneal muscles. All procedures were performed by a single fellowship-trained foot and ankle orthopedic surgeon using the same technique and post-operative protocol detailed below. All patients that met inclusion criteria were included in the study.

Exclusion criteria include a follow-up of less than 12 months, previous foot and ankle surgery, previous ankle fractures or concomitant arthritis of the ankle, presence of intra-articular pathology (loose bodies, osteochondral defects), collagen disorders, joint hypermobility syndromes, and varus/valgus deformities of the ankle.

Pre-operative examination, weight bearing radiographs, and magnetic resonance images (MRIs) were obtained for each patient that were utilized in the initial evaluation of the patient and diagnosis of chronic ankle instability along with assessments for exclusion criteria.

### 2.1. Primary Outcome

Patients were assessed pre- and post-operatively at their last follow-up examination using the Foot Function Index (FFI) score. The FFI is a patient-reported outcome tool utilized to measure the impact of pathology on function [20]. The tool has been previously validated and translated into multiple languages and used worldwide for the assessment of foot and ankle pathology [21,22,23,24]. Since its inception, there have been multiple modifications of the tool from the original form [24,25]. The version utilized in this study is the validated form modified by Venditto et al. [26]. It utilizes three sub-scales that include pain, disability, and activity restriction. Each sub-scale has multiple scoring items measured on a numeric scoring system from 1 to 10. The pain, disability, and activity restriction sub-scales have 5, 9, and 3 items, respectively. Scoring for the tool is from 0 to 100 on each sub-scale, and scores are then aggregated for a total score, with higher scores related to worse function.

### 2.2. Secondary Outcomes

Post-operative complications were recorded at regularly scheduled follow-up appointments based on the surgeon’s standard post-operative visit protocol. Additionally, the patients were given a simplified satisfaction questionnaire asking if they would undergo surgery again under the same clinical conditions at their last follow-up visit.

### 2.3. Bias

The standard biases for retrospective studies of selection bias, missing data, and loss to follow-up were applicable. There were no missing data points and no loss to follow-up for all patients. Unknown or unmeasured confounders inevitably remain due to the retrospective nature of the study.

### 2.4. Operative Technique

**Figure 1:**
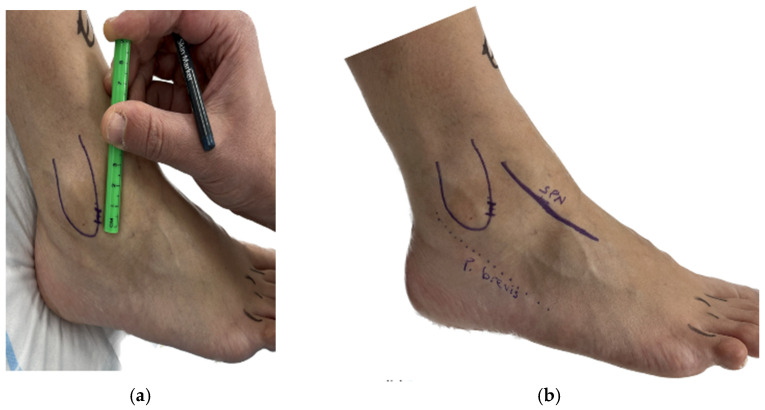
(**a**) Skin landmark of fibula and surgical incision. (**b**) Anatomic landmarks: Superficial peroneal nerve (SPN), distal fibula (unlabeled), and peroneus brevis (P. Brevis).

The procedure was performed under conscious sedation with the use of regional anesthesia. The patient was positioned supine without a tourniquet. A towel bump was placed under the ipsilateral hip to internally rotate the lower leg. Anatomic landmarks, including the superficial peroneal nerve, distal fibula, and peroneal tendon, were located pre-operatively (Figure 1).

**Figure 2:**
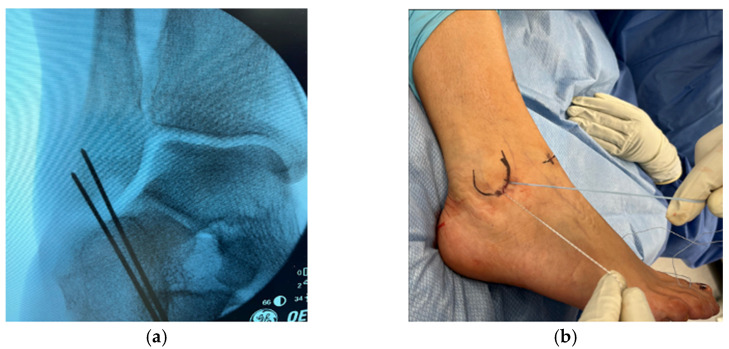
(**a**) Positioning of k-wires under fluoroscopic guidance. (**b**) Suture anchors at the midpoint of the ATFL and CFL.

Two guidewires were advanced under fluoroscopic guidance at the footprint of the ATFL. The wires were spaced 1 cm apart. The skin was incised over the wires, and the latter were then overdrilled, followed by insertion of a 3 mm BioComposite suture anchor (Arthrobrostrom, Arthex, Naples, FL, USA) (Figure 2).

**Figure 3:**
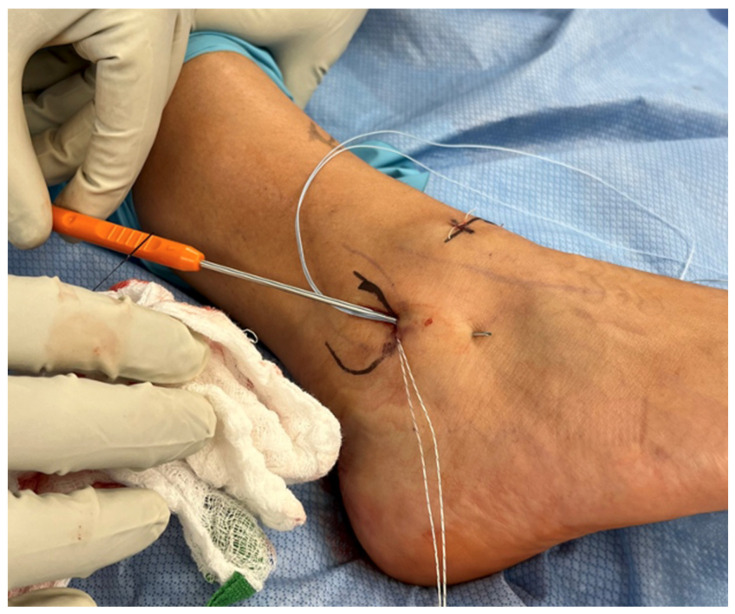
Sutures transported using a curved sharp-tipped suture passer.

A curved sharp-tipped suture passer was utilized to pass both suture ends from each anchor to the distal edge of the inferior extensor retinaculum (Figure 3). The suture was passed 1.5 cm from the distal apex of the fibula with the ankle in a neutral position, ensuring that the suture passer and sutures were within the “safe zone” of the inferior extensor retinaculum and that sufficient portions of the retinaculum were grasped [10]. Passage of these sets of sutures in this manner secured them through capsule and inferior extensor retinaculum.

**Figure 4:**
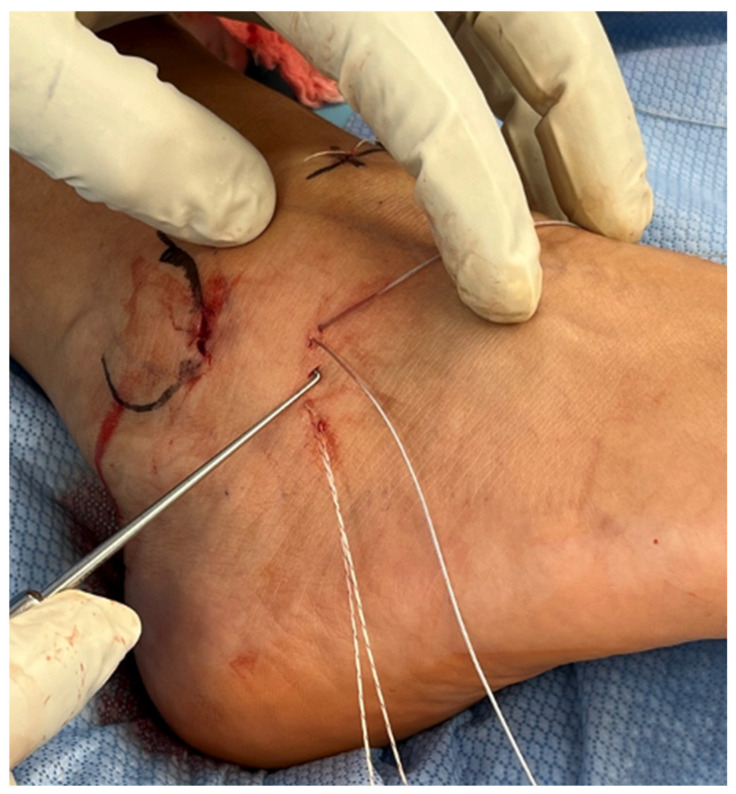
Transporting the sutures subcutaneously using an arthroscopic hook.

An incision of approximately 2 mm was made at the midpoint between the two sets of sutures. A small arthroscopic hook was used through this incision to draw the sutures subcutaneously out of the incision (Figure 4).

While maintaining the foot in neutral dorsiflexion and maximal eversion, the sutures were tied. Suture limbs were cut flush to the knot after to avoid irritation of the superficial tissues. All incisions were then closed with an absorbable suture, and a sterile dressing was applied.

### 2.5. Post-Operative Management

The ankle was protected in a CAM boot post-operatively. Patients were allowed to immediately place their full weight on the operative extremity. Active ankle range of motion was initiated two weeks after surgery. At six weeks post-operatively, the patient was allowed to wear a comfortable shoe and start physical therapy for muscle strengthening and proprioception. Return to any sports without restriction was allowed as tolerated 3 months post-operatively.

### 2.6. Statistical Analysis

Data analysis was performed using Stata/MP 16.1 (College Station, TX, USA). Descriptive statistical analysis was performed on all variables. Frequency and percentages were obtained for categorical variables. Mean, standard deviation (SD), and ranges were obtained for continuous variables. A paired t-test was used to analyze quantitative variables, including the difference between pre- and post-operative Foot Function Index (FFI) scores. The Fisher exact test was used to evaluate association among categorical variables. A *p*-value ≤ 0.05 was statistically significant.

## 3. Results

In total, 54 patients (32 males (59%) and 22 females (41%)) were included in the analysis after undergoing unilateral surgery. The mean age at the time of surgery was 33.7 years (range 19–57 years). The mean follow-up was 32.1 months, SD 14.3 (range 12–60 months), in the entire cohort. There was no loss to follow-up and no missing data variables for all patients.

### 3.1. Primary Outcome Measure

The mean total FFI score significantly improved from 54.7 (SD 4.1) pre-operatively to 10.4 (SD 1.9) post-operatively (*p* < 0.001). See Table 1 for sub-scales.

### 3.2. Secondary Outcome Measures

There was one post-operative complication. A patient required open revision with allograft reconstruction following a fall on the operative leg at 2.5 months post-operatively. The overall rate of satisfaction after surgery was 98.1% (53/54). One patient was dissatisfied with the procedure due to excessive ankle stiffness. All patients returned to their previous activity level. There were no other bone or soft tissue complications, no superficial or deep infections and no superficial peroneal nerve (SPN) injuries.

## 4. Discussion

The open Brostrom procedure was described in 1966 with a multitude of subsequent modifications, specifically the Brostrom–Gould procedure, as well as the development of arthroscopic techniques. The literature has reported satisfactory outcomes utilizing the open procedure throughout its initial report and modification [27,28]. More recently, some studies have demonstrated evidence of the superiority of arthroscopic techniques [7,18,29,30,31]. Nakasa et al. assessed clinical outcomes of arthroscopic versus open repair in 63 ankles and found superior AOFAS scores in arthroscopic repair at final follow-up [30]. Wang et al. found improved clinical outcome scores at the six-month follow-up when comparing arthroscopic to open repair in 99 patients [31]. Hou et al. compared arthroscopic to open repair in 70 patients and found faster return to sport and better patient-reported outcome scores at three and six months post-operatively. Notably, there was no difference in outcome scores at 1 and 2 years in the same study [18]. In a longer follow-up time period, Guelfi et al. followed 90 patients for an average of 4.8 years who underwent either open or all-inside arthroscopic repair. While both produced excellent clinical outcomes, they found significantly improved FFI scores at 5 years in the arthroscopic group [7]. Additionally, in a large meta-analysis composed of 408 patients in eight studies, Attia et al. found that arthroscopic repair had faster return to weight bearing and superior post-operative AOFAS and VAS scores [29].

Soft tissue complications are of specific concern in lateral ankle surgery, and the literature further supports the use of arthroscopic techniques, with studies reporting superior outcomes for wound-related complications in arthroscopic Brostrom procedures. Wound complications have been reported at significantly lower rates compared to those seen with traditional open repairs; however, no differences in overall complication rates or nerve complication rates have been reported [8,9].

As the literature on non-arthroscopic minimally invasive Brostrom techniques is sparse, the most relevant comparisons for our technique are all-arthroscopic and arthroscopic-assisted techniques as they similarly benefit from minimal soft tissue disruption. The present study demonstrates that the minimally invasive percutaneous Brostrom technique is safe and effective and can be performed without the use of arthroscopy unless there is concern for intra-articular pathology [11]. If there is any suspicion of intra-articular lesions, diagnostic arthroscopy is recommended.

Overall, this study presents significant clinical implications for the treatment of chronic lateral ankle instability. The demonstrated advantages include reductions in soft tissue involvement, leading to less post-operative pain and theorized faster functional recovery. Additionally, the simplicity of the technique allows surgeons without advanced arthroscopic skills to complete the procedure, increasing the availability of options in low-resource areas. High patient satisfaction (98.1% satisfaction rate), a low rate of complications, with no infections or nerve injuries, and a single revision were reported, consistent with outcomes of previously establish techniques.

When considering the current results (98.1% satisfaction rate) in the context of arthroscopic results reported previously, which range from 79.0% to 100% depending on the use of open or arthroscopic techniques, this reported technique performs well [32]. Regarding complications in the current study, only 1 patient out of 54 reported ankle stiffness. Historically, this has been more commonly described in patients undergoing allograft reconstruction or non-anatomic repair [33,34].

Notably the present study has limitations. These include retrospective design, a relatively small cohort, and the lack of control group. Additionally, this technique cannot address intra-articular pathology, so the surgeon must appropriately indicate patients for the procedure. Another limitation to the study is the lack of a specific objective assessment for instability. Even with this limitation, the clinical evaluation still provides valuable data that demonstrate the technique’s effectiveness. Despite these limitations, the results suggest that there is high satisfaction, post-operative improvement, and limited complications associated with the minimally invasive non-arthroscopic technique in the appropriate patient.

The results of this study are promising, as the described technique is similar to the arthroscopic procedure described by Acevedo et al. [10]; however, the advantage of the current, existing technique is that it can be performed by those unfamiliar or uncomfortable with ankle arthroscopy [19].

## 5. Conclusions

The minimally invasive percutaneous Brostrom procedure is safe and effective for the treatment of chronic lateral ankle instability without intra-articular pathology. Patients report significant post-operative improvement and overall satisfaction with outcomes similar to those of other established techniques such as the arthroscopic and open Brostrom technique.

The current results are promising. Further studies with larger cohorts directly comparing techniques, such as open and arthroscopic surgery versus percutaneous surgery, would be beneficial in expanding the minimally invasive ankle surgery literature.

## Figures and Tables

**Table 1:** Foot function index (Sub-scale pre- and post-operative scores).

	FFI—Pain	FFI—Disability	FFI—Activity Limitation	FFI—Total
Pre-Op Score	20.5	16.3	17.9	54.7 ^1^
Post-Op Score	2.9	3.9	3.6	10.4 ^1^

^1^ Total FFI was significantly improved from pre- to post-operative time points. Improvements were observed in all domains of the FFI for the cohort.

## Data Availability

The de-identified data presented in this study are available on request from the corresponding author due to HIPAA and IRB requirements.
